# Supplementary figures and images for: Organic matter processing by heterotrophic bacterioplankton in a large tropical river: Relating elemental composition and potential carbon mineralization
Source: PLoS One. 2024 Nov 11;19(11):e0311750. doi: 10.1371/journal.pone.0311750 (PMC11554041; doi:10.1371/journal.pone.0311750)

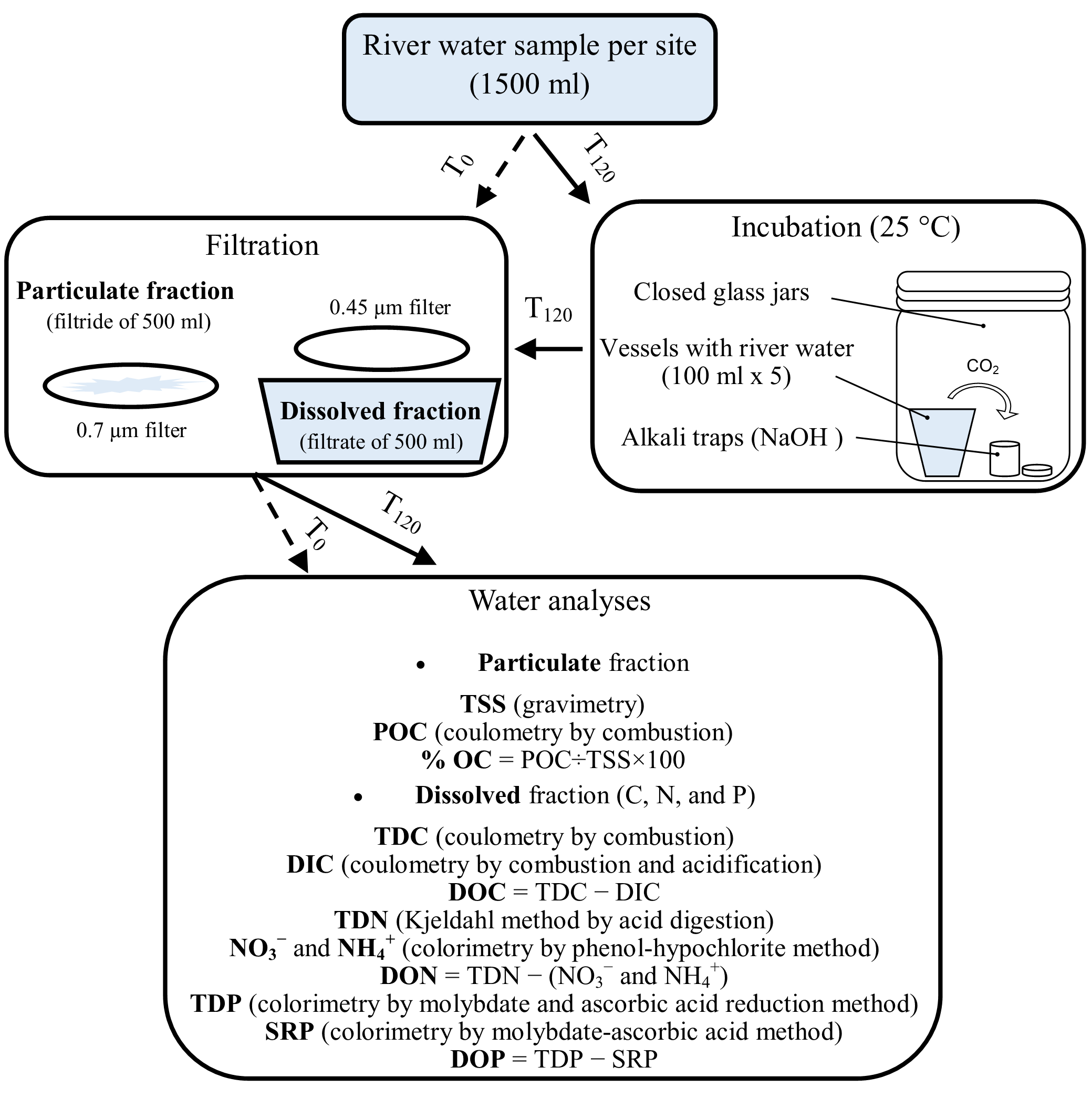

Supplement: S1 Fig — At each season (dry and rainy) and in each site (Lacantún, Balancán, and Centla), one river water sample batch was used to determine initial water conditions (dotted arrow; referred to as T0 in the main text). The remaining sample was used to perform the incubation experiment and determine the final water conditions (straight arrow; referred to as T120 in the main text). (TIF) [file pone.0311750.s001.tif]
