# Supplementary material for: Organic matter processing by heterotrophic bacterioplankton in a large tropical river: Relating elemental composition and potential carbon mineralization
Source: PLoS One. 2024 Nov 11;19(11):e0311750. doi: 10.1371/journal.pone.0311750 (PMC11554041; doi:10.1371/journal.pone.0311750)
Supplement: S1 Table — (DOCX) [file pone.0311750.s002.docx]

**S1 Table. Evaluated physical and chemical variables at the incubation experiment's initial (T_0_) and final (T_120_) times.**

|  | Variable | Abbrev. | Method | Reference |
| --- | --- | --- | --- | --- |
| Particulate fraction | Total suspended solids | TSS | Gravimetry | APHA 2017 |
|  | Particulate organic carbon | POC | Combustion, coulometry | Huffman 1977; APHA 2018 |
|  | Percentage of organic carbon | %OC. | %OC = (POC / TSS) x 100 | Ittekkot 1988; Meybeck 1993 |
| Dissolved fraction | Total dissolved carbon | TDC | Combustion, coulometry | Huffman 1977; APHA 2018 |
|  | Dissolved inorganic carbon | DIC | Combustion, coulometry after acidification | Huffman 1977; APHA 2018 |
|  | Dissolved organic carbon | DOC | DOC = TDC − DIC | Huffman 1977; APHA 2018 |
|  | Total dissolved nitrogen | TDN | Kjeldahl, after acid digestion | Bremner 1996 |
|  | Nitrate | NO_3_^−^ | Colorimetry, phenol–hypochlorite | Mulvaney 1996 |
|  | Ammonium | NH_4_^+^ | Colorimetry, phenol–hypochlorite | Mulvaney 1996 |
|  | Dissolved organic nitrogen | DON | DON = TDN −(NO_3_^−^ + NH_4_^+^) |  |
|  | Total dissolved phosphorous | TDP | Colorimetry, molybdate, and ascorbic acid reduction | Murphy and Riley 1962 |
|  | Soluble reactive phosphorous | SRP | Colorimetry, molybdate, and ascorbic acid reduction | Murphy and Riley 1962 |
|  | Dissolved organic phosphorous | DOP | DOP = TDP – SRP |  |
