# Supplementary material for: Organic matter processing by heterotrophic bacterioplankton in a large tropical river: Relating elemental composition and potential carbon mineralization
Source: PLoS One. 2024 Nov 11;19(11):e0311750. doi: 10.1371/journal.pone.0311750 (PMC11554041; doi:10.1371/journal.pone.0311750)
Supplement: S2 Table — (DOCX) [file pone.0311750.s003.docx]

**S2 Table. Changes of particulate and dissolved nutrients after incubating water samples from Lacantún, Balancán, and Centla in two contrasting seasons.**

|  |  | Dry season | | | Rainy season | | |
| --- | --- | --- | --- | --- | --- | --- | --- |
|  |  | Lacantún | Balancán | Centla | Lacantún | Balancán | Centla |
| TSS | T_0_ | 3.3 | 18.3 | 5.0 | 36.7 | 50.0 | 27.5 |
|  | T_120_ | 11.6 | 6.1 | 1.7 | 34.7 | 40.9 | 22.8 |
|  | Δ | 8.3 | −12.22 | −3.3 | −2.0 | −9.1 | −4.7 |
|  | Δ% | 248 | −67 | −67 | −6 | −18 | −17 |
| POC | T_0_ | 0.35 | 2.03 | 0.46 | 0.86 | 1.80 | 0.55 |
|  | T_120_ | 2.35 | 1.06 | 0.29 | 1.69 | 2.16 | 0.75 |
|  | Δ | 2.00 | −0.97 | −0.17 | 0.83 | 0.36 | 0.20 |
|  | Δ% | 582 | −48 | −36 | 101 | 21 | 37 |
| %OC | T_0_ | 10 | 11 | 9 | 2 | 4 | 2 |
|  | T_120_ | 20 | 17 | 18 | 5 | 5 | 3 |
|  | Δ | 10 | 6 | 9 | 3 | 2 | 1 |
|  | Δ% | 96 | 57 | 93 | 113 | 51 | 65 |
| DIC | T_0_ | 27,310 | 33,790 | 30,040 | 30,660 | 28,910 | 31,850 |
|  | T_120_ | 15,040 (320) | 15,810 (1,050) | 20,760 (480) | 18,980 (400) | 21,730 (960) | 26,910 (820) |
|  | Δ | −12,270 (320) | −17,920 (1,050) | −9,290 (480) | −11,680 (400) | −7,180 (960) | −4,940 (820) |
|  | Δ% | −45 (1) | −53 (3) | −31 (2) | −38 (1) | −25 (3) | −16 (2) |
| DOC | T_0_ | 22,580 | 20,930 | 12,620 | 7,260 | 8,650 | 8,030 |
|  | T_120_ | 8,360 (1,110) | 7,410 (2,160) | 3,350 (1,790) | 3,310 (1,380) | 2,540 (190) | 3,880 (1,710) |
|  | Δ | −14,220 (1,110) | −13,520 (2,160) | −9,270 (1,790) | −3,950 (1,380) | −6,110 (190) | −4,160 (1,710) |
|  | Δ% | −63 (5) | −65 (10) | −73 (14) | −54 (19) | −71 (2) | −52 (21) |
| NO_3_^−^ | T_0_ | 18 | 12 | * | 5 | 30 | 82 |
|  | T_120_ | 18 (8) | 19 | * | * | * | 65 (9) |
|  | Δ | 1 (8) | 7 | * | * | * | −17 (9) |
|  | Δ% | 3 (45) | 65 | * | * | * | −21 (11) |
| NH_4_^+^ | T_0_ | 9 | * | 14 | 124 | 137 | 181 |
|  | T_120_ | 27 (8) | 6 (4) | 20 | 8 (10) | 10 (9) | 112 (48) |
|  | Δ | 18 (8) | * | 6 | −116 (10) | −127 (9) | −69 (48) |
|  | Δ% | 196 (86) | * | 43 | −94 (8) | −93 (7) | −38 (26) |
| DON | T_0_ | 5 | 40 | 99 | 66 | 46 | 5 |
|  | T_120_ | 14 (13) | 73 (28) | 42 | 197 (13) | 271 (33) | 104 (17) |
|  | Δ | 9 (13) | 33 (28) | −57 | 131 (13) | 225 (33) | 99 (17) |
|  | Δ% | 200 (283) | 85 (72) | −58 | 198 (19) | 488 (71) | 1,970 (336) |
| SRP | T_0_ | * | * | * | 2 | 2 | 21 |
|  | T_120_ | * | * | * | * | * | 4 (1) |
|  | Δ | * | * | * | * | * | −17 (1) |
|  | Δ% | * | * | * | * | * | −83 (3) |
| DOP | T_0_ | 4 | 28 | 327 | 22 | 14 | * |
|  | T_120_ | 34 (10) | 25 (5) | 33 (6) | 24 | 6 (6) | 26 (13) |
|  | Δ | 30 (10) | −3 (5) | −294 (6) | 2 | −8 (6) | * |
|  | Δ% | 744 (254) | −10 (17) | −90 (2) | 9 | −61 (45) | * |
| C/N | T_0_ | 5,018 | 530 | 127 | 110 | 188 | 1,606 |
|  | T_120_ | 1,131 (1002) | 93 (36) | 49 | 19 (9) | 9 (2) | 30 (9) |
|  | Δ | −3,886 (1002) | −437 (36) | −79 | −91 (9) | −179 (2) | −1,576 (9) |
|  | Δ% | −77 (20) | −83 (7) | −63 | −83 (8) | −95 (1) | −98 (1) |
| C/P | T_0_ | 5,645 | 748 | 39 | 330 | 618 | * |
|  | T_120_ | 237 (52) | 298 (28) | 110 (72) | 147 | 463 (45) | 112 (56) |
|  | Δ | −5,408 (52) | −449 (28) | 72 (72) | −183 | −155 (45) | * |
|  | Δ% | −96 (1) | −60 (4) | 186 (187) | −56 | −25 (7) | * |
| N/P | T_0_ | 1.1 | 1.4 | 0.3 | 3.0 | 3.3 | * |
|  | T_120_ | 0.3 (0.3) | 4.8 (3.0) | 1.2 | 8.2 | 24.6 (2.6) | 6.0 (4.2) |
|  | Δ | −0.8 (0.3) | 3.3 (3.0) | 0.9 | 5.2 | 21.3 (2.6) | * |
|  | Δ% | −71 (28) | 237 (214) | 303 | 174 | 647 (80) | * |

Values represent the mean (standard deviation) and deltas (absolute, Δ, and relative, Δ%; regarding T_0_) of the initial (T_0_) and final (T_120_; 120 h at 25 °C) incubation conditions. TSS (mg l^−1^): total suspended solids; POC (mg l^−1^): particulate organic carbon; %OC (%): percentage of organic carbon in TSS; DIC (µg l^−1^): dissolved inorganic carbon; DOC (µg l^−1^): dissolved organic carbon; NO_3_^−^ (µg l^−1^): nitrates; NH_4_^+^ (µg l^−1^): ammonium; DON (µg l^−1^): dissolved organic nitrogen; SRP (µg l^−1^): soluble reactive phosphorus; DOP (µg l^−1^): dissolved organic phosphorus. *: values below the detection limit.
