# Supplementary material for: Organic matter processing by heterotrophic bacterioplankton in a large tropical river: Relating elemental composition and potential carbon mineralization
Source: PLoS One. 2024 Nov 11;19(11):e0311750. doi: 10.1371/journal.pone.0311750 (PMC11554041; doi:10.1371/journal.pone.0311750)
Supplement: S3 Table — (DOCX) [file pone.0311750.s004.docx]

**S3 Table. Slope and intercept of linear regressions (df: 1 and 4) between log-transformed C/N/P ratios at T_120_ and C/N/P ratios at T_0_ of the dissolved organic matter.**

| Relationship | Intercept | Slope | r^2^ | F | P value |
| --- | --- | --- | --- | --- | --- |
| DOC/DON_T120_ vs DOC/DON_T0_ | 3.0 ± 0.5 | 7 · 10^−4^ ± 2 · 10^−4^ | 0.75 | 12.1 | 0.02 |
| ΔDOC/DON_T120_ vs DOC/DON_T0_ | 8.9 ± 0.4 | −1.6 · 10^−3^ ± 2 · 10^−4^ | 0.94 | 65.5 | <0.01 |
| DOC/DOP_T120_ vs DOC/DOP_T0_ | 5.2 ± 0.3 | 5 · 10^−5^ ± 2 · 10^−4^ | 0.05 | 0.2 | 0.68 |
| ΔDOC/DOP_T120_ vs DOC/DOP_T0_ | 9.3 ± 0.2 | −1.6 · 10^−3^ ± 5 · 10^−5^ | 0.98 | 310.7 | <0.01 |
| DON/DOP_T120_ vs DON/DOP_T0_ | −0.8 ± 0.7 | 1.0 ± 0.4 | 0.64 | 7.1 | 0.05 |
| ΔDON/DOP_T120_ vs DON/DOP_T0_ | 0.3 ± 0.6 | 0.6 ± 0.3 | 0.61 | 6.2 | 0.06 |
