# Supplementary material for: Organic matter processing by heterotrophic bacterioplankton in a large tropical river: Relating elemental composition and potential carbon mineralization
Source: PLoS One. 2024 Nov 11;19(11):e0311750. doi: 10.1371/journal.pone.0311750 (PMC11554041; doi:10.1371/journal.pone.0311750)
Supplement: S4 Table — (DOCX) [file pone.0311750.s005.docx]

**S4 Table. Central tendency values and of the potential C mineralization (CO_2_–C) after incubation (T_120_ at 25 °C) of the water samples from the Usumacinta River sites [Lacantún, Balancán, and Centla] in the dry and rainy seasons.**

| Season | Site | C–CO_2_ (mg l^−1^ day^−1^) | | | Source of variation | |
| --- | --- | --- | --- | --- | --- | --- |
|  |  | x̄ | σ | n | Site | Season |
| Dry season | Lacantún | 12.0 | 4.5 | 5 | A | a |
|  | Balancán | 14.8 | 2.7 | 5 | A | a |
|  | Centla | 6.3 | 0.7 | 5 | B | a |
| Rainy season | Lacantún | 7.9 | 7.9 | 5 | A | a |
|  | Balancán | 5.5 | 3.5 | 5 | A | b |
|  | Centla | 7.0 | 4.0 | 5 | A | a |

x̄: sample mean; σ: sample standard deviation; *x̄*: sample median; n: sample size. Different uppercase letters show samples significantly different between sites within seasons, while different lowercase letters show samples significantly different between seasons within sites according to mixed–design ANOVA.
