# Supplementary material for: Organic matter processing by heterotrophic bacterioplankton in a large tropical river: Relating elemental composition and potential carbon mineralization
Source: PLoS One. 2024 Nov 11;19(11):e0311750. doi: 10.1371/journal.pone.0311750 (PMC11554041; doi:10.1371/journal.pone.0311750)
Supplement: Attachment — Submitted filename: reviewers conmments.docx [file pone.0311750.s006.docx]

I think this paper have demonstrated a well and detail work for the carbon and nitrogen change in POM intersecting the different condition of the upstream and downstream flows and the seasons ( dry and rainy). And the incubation was used to detect the function of microbial mineralization. This is a good combination to the biological and physical/ meteorology, but I think the research difficulty is not too high from From the perspective of method. And the conclusion is not too breakthrough discoveries or impressive view.

In addition, the Organic matter processing by potamoplankton can not be found in the research except the filter process with 0.7μm Pore diameter of filter membrane to filter the particulate fraction. Maybe I can not grasp your method because my academic level is limited, so i think the connection between the potamoplankton and the organic matter processing should be be analyzed in the paper.
