# Supplementary material for: Organic matter processing by heterotrophic bacterioplankton in a large tropical river: Relating elemental composition and potential carbon mineralization
Source: PLoS One. 2024 Nov 11;19(11):e0311750. doi: 10.1371/journal.pone.0311750 (PMC11554041; doi:10.1371/journal.pone.0311750)
Supplement: Attachment — Submitted filename: description.docx [file pone.0311750.s008.docx]

**“Organic matter processing by heterotrophic bacterioplankton in a large tropical river: relating elemental composition and potential carbon mineralization”**

**The content of manuscript is promising. Though It need the improvement in the methods section.**

**Author should have used few advanced techniques to explain the study.**

**The English of manuscript is need to be improved.**

**However, the study of this manuscript should be published, since it has explained the environment impact of the tropical river.**
